# Guayule Natural Rubber Latex and Bi_2_O_3_ Films for X-ray Attenuating Medical Gloves

**DOI:** 10.3390/ma15031184

**Published:** 2022-02-04

**Authors:** David A. Ramirez Cadavid, Rick R. Layman, Thomas Nishino, J. Lauren Slutzky, Zhenyu Li, Katrina Cornish

**Affiliations:** 1 Department of Food, Agricultural and Biological Engineering, The Ohio State University, 1680 Madison Avenue, Wooster, OH 44691, USA; ramirezcadavid.1@osu.edu (D.A.R.C.); slutzky.1@buckeyemail.osu.edu (J.L.S.); 2 Department of Imaging Physics, MD Anderson Cancer Center, The University of Texas, 1400 Pressler Street, Houston, TX 77030, USA; RRLayman@mdanderson.org (R.R.L.); TNishino@mdanderson.org (T.N.); 3 EnergyEne Inc., 5659 Canaan Center Road, Wooster, OH 44691, USA; stevenzhenyuli@gmail.com; 4 Department of Horticulture and Crop Science, The Ohio State University, 1680 Madison Avenue, Wooster, OH 44691, USA

**Keywords:** natural rubber, radiation protection, attenuation properties, rubber composites, tensile properties

## Abstract

Existing natural latex radiation-attenuating gloves (RAGs) contain a high loading of radiation attenuation filler that reduces their mechanical properties to below Food and Drug Administration (FDA) medical glove requirements. RAGs are commonly formulated using Hevea natural rubber latex and lead-based fillers. The former can cause life-threatening allergic responses and the latter are known for their toxicity. In this work, a new lead-free RAG formulation based on circumallergenic guayule natural rubber latex (GNRL) and non-toxic radiation attenuation filler bismuth trioxide (Bi_2_O_3_) was developed. GNRL films with Bi_2_O_3_ loadings ranging from 0 to 300 PHR at different thicknesses were prepared. Radiation attenuation efficiencies (AE) at 60, 80, 100, and 120 kVp were determined and attenuation isocontour curves predicted film thickness and Bi_2_O_3_ loading required to meet or exceed the radiation attenuation requirements of ASTM D7866 and commercial RAGs. Optimal curing conditions for GNRL/Bi_2_O_3_ films with 150 PHR Bi_2_O_3_ were investigated by varying curing temperatures and time from 87 °C to 96 °C and 65 min to 90 min, respectively. In general, as the loading of the filler increased, the density of the films increased while the thickness decreased. GNRL/Bi_2_O_3_ films with 150 PHR Bi_2_O_3_ and 0.27 mm provided 5% more AE than RAG market average attenuation at the same thickness. The films with 150 PHR Bi_2_O_3_ cured under near-optimal conditions (90 °C/85 min, and 87 °C/65 min) met both the radiation attenuation standard (ASTM D7866) and the natural latex surgeon and examination glove standards (ASTM D3577 and D3578, respectively). Thus, gloves made using our formulations and protocols demonstrated potential to meet and surpass medical natural latex glove standards, offer a single product for both infection control and radiation protection instead of double-gloving, provide a greater degree of comfort to the user, and simultaneously reduce contact reactions and eliminate potential latex allergic reaction.

## 1. Introduction

Radiographic imaging is used in a wide variety of medical examinations and procedures to diagnose or treat illnesses [1,2,3]. As technological capabilities have advanced, radiographic imaging has been on the rise in the United States [4,5,6,7]. Image-guided interventional procedures doubled from 2001 to 2010 compared to the previous decade of 1991–2000 [7]. Likewise, X-ray imaging exams, such as computed tomography (CT), increased by 20% from 2006 to 2016 [6], with an annual increase of 1% to 5% in the same period [4]. This trend is anticipated to advance at a rapid rate with improved imaging-based technology, increased recognition and implementation of interventional radiology services, and hybrid operating rooms that improve patient outcomes and reduce patient hospitalization [7].

Physicians performing interventions with radiographic equipment assume a unique occupational risk compared to other imaging-based specialties as they are required to be in the procedure room where radiation is emitted. It is well-established that radiation exposure to an individual is inversely proportional to the square distance from the X-ray source; thus, radiation exposure significantly increases the closer the worker is to the X-ray source. This exposure poses a risk to the health care workers (HCWs), as medical procedures often require them to work close to the patient and radiation source. In some instances, the HCW is required to place their hands within the direct radiation field [1,8]. The consequences of the increased radiation exposure to the HCWs are stochastic effects leading to possible cancer induction or deterministic effects that result in skin burns or cataracts [3]. The Nuclear Regulatory Commission (NRC) and local state governing bodies require all medical institutions that utilize radiation-generating equipment to establish a radiation safety program. The general goal of these programs is to monitor and ensure that the cumulative exposure to the skin and extremities does not exceed 500 mSv/year [3]. In practice, most institutions establish thresholds at 1/10th of the maximum permitted dose for each HCW to maintain radiation exposures at “As Low As Reasonably Achievable (ALARA)” levels [8,9,10]. Required radiation protective garments, such as aprons, gloves, and eyewear, as well as protective barriers, are provided to the HCWs using radiation. In addition, radiation monitors are provided in order to measure the cumulative radiation exposure to the body and hands to ensure that ALARA levels are not exceeded.

Radiation-attenuating gloves (RAGs) are critical, especially when the HCWs must place their hands directly in the radiation field. Wearing RAGs can reduce radiation hand exposure by about 50% [11], thus minimizing the potential of stochastic or deterministic effects from chronic radiation exposure. The FDA considers the RAG a personnel protective shield intended to prevent unnecessary exposure to radiation during radiological procedures by providing an attenuating barrier [12]. However, according to the FDA, RAGs often do not provide a sufficient barrier against potentially infectious materials or contaminants; therefore, double gloving RAGs with medical gloves—examination or surgical—is required to fully protect health care workers [13,14]. The main issue with RAGs is the poor mechanical performance of gloves due to the amount of attenuating filler, which prevents many of them from achieving medical glove Type 1 (natural) and Type 2 (synthetic) performance standards, specified in ASTM D3577 [15] and D3578 [16] for surgical and examination gloves, respectively. Despite the protection offered by RAGs, they are not regularly worn by health specialists over a medical glove since they are thicker and heavier than regular medical gloves, thus decreasing tactile sensation, hand dexterity and fine motor control in the fingers, and causing hand fatigue [10].

RAGs are formulated using different elastomers and radiation attenuation fillers. The preferred elastomer for RAGs is Hevea natural rubber latex (HNRL) (harvested from the tropical rubber tree, *Hevea brasiliensis*) because of its higher filler capacity and tensile properties compared to synthetic elastomers [17]. HNRL gloves confer more fine finger control and sensitivity to touch than synthetic gloves [18]. However, HNRL contains proteins that can cause life-threatening, systemic allergic responses, associated with significant morbidity and potential mortality among sensitized HCWs and patients [19,20].

Guayule natural rubber latex (GNRL) is an alternative circumallergenic natural elastomer mechanically extracted from the desert shrub *Parthenium argentatum*. GNRL contains 1% of the protein in HNRL, and none cross-react with antibodies raised against Hevea latex proteins [21]. GNRL’s low protein, high fatty acid content and linear rubber polymers make it softer and more elastic than Hevea rubber [22,23] and, at the same time, enable a high filler loading while maintaining outstanding physical properties [3]. Additionally, dipped products of GNRL, such as gloves and condoms, have been proven to provide an effective barrier against the transmission of human viruses [24]. These characteristics make GNRL particularly attractive in the production of elastomeric medical products.

Lead and a handful of heavy elements with high atomic numbers (Z) are known to effectively absorb high-energy radiation, and hence, they are considered shielding materials [25,26]. Lead (Pb, Z = 82) and its derivatives have traditionally served as attenuation fillers because of the low cost, high density, and effectiveness in reducing the ionizing radiation. However, lead is an acutely toxic metal, and extended exposure may lead to severe health issues, including neurological disorders, kidney failure, and reductions in hemoglobin and red blood cells [27,28]. Therefore, it has been essential to limit the use of lead in general and to study other less toxic metals and synthesized materials, such as aluminum, copper, antimony, barium, gadolinium, tungsten, bismuth, and silicate glass, in the development of new radiation attenuation products and applications [13,14,25,29,30,31,32,33,34,35]. Dependence on these materials has been growing due to the increased use of and/or exposure to ionizing radiation in the industry and research in fields such as aerospace, electronics, agriculture, optics, biology, and medicine [25,36,37,38,39,40]. 

Among these materials, bismuth (Bi, Z = 83) and its derivatives have become of interest in fields of electronics, aerospace, chemistry, and medicine [29,30,31]. The high Z of bismuth makes it an effective shielding material for protection against high-energy radiation and ions, and thus, it is being used in radiation attenuation applications [25,41,42,43,44]. In contrast with lead-based radiation attenuation materials, bismuth-based materials are safer, non-toxic, and environmentally friendly and can provide the same or more effective shielding due to their comparable Z [29,30,38]. Additionally, lead-based fillers are dense and when added into polymers and elastomers regularly used for RAG, such as styrene-butadiene rubber and natural rubber, they can cause aging, embrittlement, cracking, and a decline in the mechanical properties, thus reducing the lifetime of the attenuating composites [39,45]. Therefore, Bi-based materials are a more effective alternative in the development of RAG.

Bismuth trioxide (Bi_2_O_3_) is a Bi derivative that has 89% of Bi by weight, which makes it an excellent candidate for radiation attenuation applications, including RAGs. Bi_2_O_3_ is considered safe for medical applications [46,47], and moreover, it is odorless, inexpensive, and abundant, with stable mechanical and chemical properties [48,49].

In this study, GNRL was used as an elastomer matrix for the dispersion of micro-sized Bi_2_O_3_ (D = 6.4 μm) at different concentrations. Films with different thicknesses were made using casting and dipping methods. X-ray attenuation properties at 60, 80, 100, and 120 kVp were determined and used to calculate linear attenuation coefficient. GNRL/Bi_2_O_3_ films with optimal attenuations were selected for curing optimization based on the mechanical properties. Finally, GNRL/Bi_2_O_3_ prototype gloves were made, and the mechanical properties were determined.

## 2. Materials and Methods

### 2.1. Formulation

The recipe for compounding GNRL is shown in Table 1. Ammonium hydroxide was obtained from Sigma Aldrich (St. Louis, MO, USA). Dispersions of antioxidant (Bostex 24), zinc oxide (Bostex 422), and sulfur (Bostex 378) were generously donated by Akron Dispersions (Akron, OH, USA). A xanthate-based accelerator package ZDNC and DIXP were obtained from Robinson Brothers Limited (West Bromwich, West Midlands, UK). Micro-sized attenuation filler (bismuth trioxide, Bi_2_O_3_) was sourced from Ferro Corporation (Product ID 320, particle size D = 6.4 μm, Mayfield Heights, OH, USA).

### 2.2. Dispersion and Compounding of Bi_2_O_3_ in the GNRL

The GNRL compound without attenuation filler was prepared by mixing the ingredients (Table 1) using a laboratory stirrer Caframo BDC2002 (Caframo limited, Georgian Bluffs, ON, Canada) at 200–1000 rpm. 

**Figure 1:**
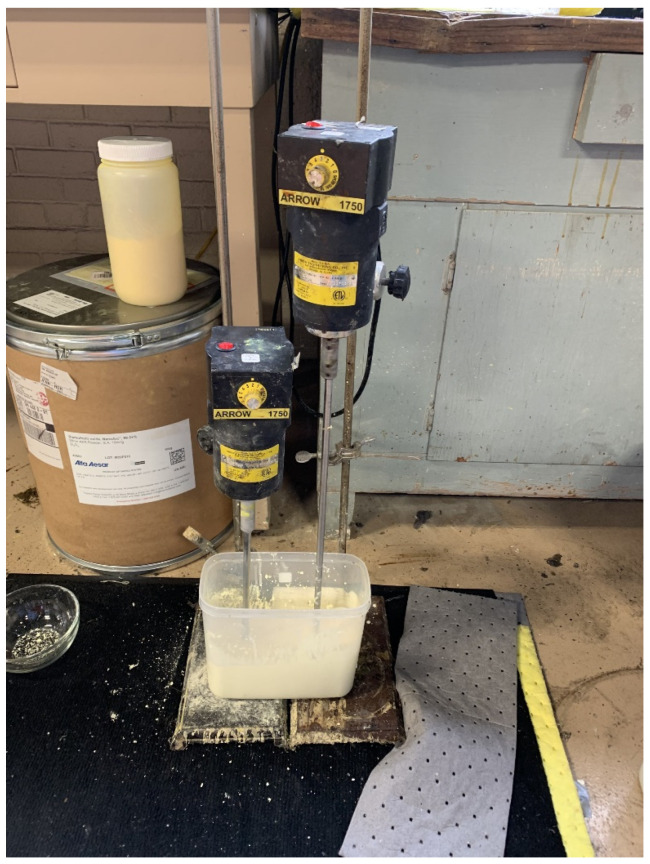
Compounding of micro-Bi_2_O_3_ in the GNRL.

Adding Bi_2_O_3_ powder directly into GNRL created an uneven dispersion of the two materials due to the agglomeration of the filler particles. Obtaining a uniform distribution of micro-Bi_2_O_3_ in GNRL was facilitated by first dispersing Bi_2_O_3_ in water and then mixing the Bi_2_O_3_/water into the GNRL (Figure 1). 

Micro-Bi_2_O_3_ was dispersed into the double-deionized water at a loading of up to 90% using a high-speed laboratory mixer, Fisher brand RealTorqueDigital (Thermo Fisher Scientific, Waltham, MA, USA), at >1500 rpm. The high loading of Bi_2_O_3_/water dispersion was then readily mixed with GNRL to achieve loadings up to 900 parts of Bi_2_O_3_ per hundred of rubber (PHR) by weight (or 0.89 mass fraction). Then, 5–10 g wet weight of water-dispersed Bi_2_O_3_ was added to the GNRL and fully dispersed before subsequent additions to avoid agglomeration of the filler in the latex (Figure 1). The mixing was at a slow velocity at the beginning and then gradually increased to disperse the Bi_2_O_3_ in the latex compound evenly. Additionally, the type of agitator used facilitated the suspension of the water-dispersed Bi_2_O_3_; gate and anchor blades worked better than turbine, spiral propeller, or paddle blades. The final mixture (a suspension of Bi_2_O_3_ in GNRL) was filtered through two layers of 110 mesh (140 µm) silkscreen. Clumping was not detected at any of the loadings produced in this study (50, 100, 150, 200, 250, and 300 PHR). To eliminate air bubbles and prevent sedimentation of the Bi_2_O_3_ during storage in the dark at 4 °C, the dispersion was continually mixed at low velocity (<500 rpm) for at least 12 h.

### 2.3. Film and RA Gloves Preparation

Films of GNRL without (0 PHR) and with Bi_2_O_3_ at 50, 100, 150, 200, 250, and 300 PHR were produced either by casting or dipping.

Cast films of GNRL and GNRL/Bi_2_O_3_ were obtained at different thicknesses by varying the amount of GNRL/Bi_2_O_3_ suspension transferred into plastic Petri dishes (8.57 cm in diameter). The suspension was gently manually mixed before each sample was collected with a pipette. Each sample was slowly dispensed onto a Petri dish to avoid entrapment of air bubbles. Finally, the Petri dishes with the suspension were moved onto a leveled shelf to prevent the formation of an uneven dispersion of the attenuation filler (Bi_2_O_3_) layer. The films were cured at 50 °C for 48–60 h to allow any remaining air bubbles to escape. The films were carefully peeled from the Petri dishes, and thickness was measured using a micrometer. Triplicate samples were produced for each compound and thickness.

**Figure 2:**
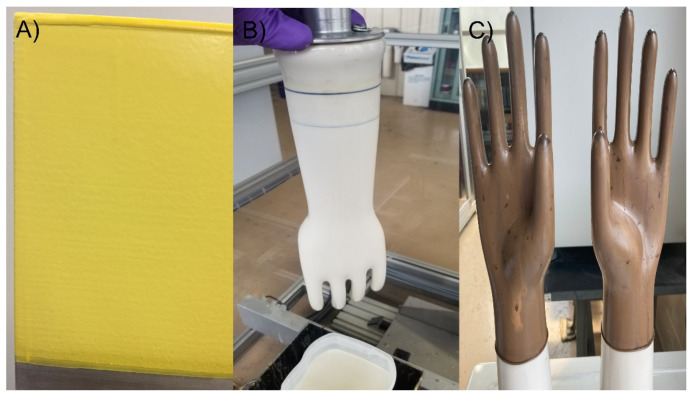
Films and RA gloves preparation. (**A**) Pre-curing of GNRL/micro-Bi_2_O_3_ film prepared by dipping; (**B**) Preparation of glove former before dipping into GNRL/micro-Bi_2_O_3_; (**C**) Post-curing RA gloves.

Films of GNRL and GNRL/Bi_2_O_3_ prepared by dipping were obtained using aluminum plate formers (DipTech Systems Inc., Kent, OH, USA) mounted in a Diplomat computerized latex dipper (DipTech Systems Inc.). The formers were preheated to a specific temperature and dipped into a coagulant solution (Table 2). The coagulant was dried at 70 °C for at least 20 min and then dipped into the GNRL/Bi_2_O_3_ suspension to produce uncured films (Figure 2A). The films were treated with a polyurethane coating before being fully cured by vulcanization in a curing oven (Heratherm oven, Thermo Fisher Scientific, Waltham, MA, USA). Film thicknesses were controlled by dwell time in the suspension, which ranged from 3 s to 20 s. Films for each treatment were produced in triplicate. Two film samples were made during each dipping (both sides of the plate former were coated). One of the films was used for tensile testing, and the other was used for attenuation and uniformity testing. 

Radiation attenuation gloves were made by dipping ceramic formers (DipTech Systems Inc.) mounted in a Diplomat computerized latex dipper (DipTech Systems Inc.) following the method described above for films (Figure 2B). Glove thicknesses were controlled by dwell time of 20 s in the GNRL/Bi_2_O_3_ suspension. Gloves were produced by dipping only one size (M) of surgical glove ceramic former and Bi_2_O_3_ loading of 150 PHR. The gloves were cured at 70 °C for 70 min (Figure 2C).

### 2.4. Radiation Attenuation 

**Figure 3:**
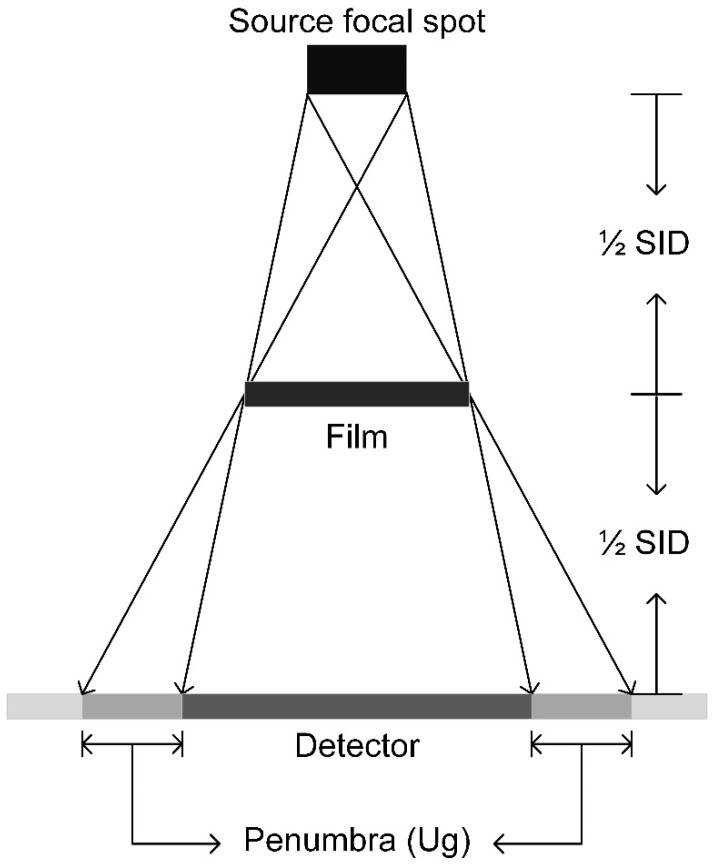
Illustration of film radiation attenuation measurement. A source-to-image distance (SID) was set at 45 cm and films were suspended with a stand at half of the SID.

X-ray attenuation properties of the films were measured at 60, 80, 100, and 120 kVp according to ASTM D7866 [50] using a calibrated digital radiography system (GE discovery XR656 Plus, GE Healthcare systems, Waukesha, WI, USA) and non-invasive radiation detection system with an ionization chamber (Radcal Accu-Gold+ and 10 × 6 − 6 chamber, Radcal Corporation, Monrovia, CA, USA). The radiography system was set up for tabletop exposures without the Bucky and using a source-to-image distance (SID) of 45 cm. The ionization chamber was placed tabletop with a lead apron underneath to prevent radiation backscatter. The radiation field was collimated to the film to ensure narrow geometry and films were suspended with a stand at half of the SID or closer to the X-ray tube source (Figure 3). Exposures were made with and without the film sample in the X-ray field. The ionization chamber exposures were recorded and reported as the percent attenuation. 

The percentage attenuation, or attenuation efficiency (AE), was calculated using the Beer–Lambert law (Equation (1)):

(1) $$I\left(d\right)=I_{0}e^{-\mu d\;}$$

where $I_{0}$ and I(d) are the peak intensities of the X-rays without and with a film of GNRL/Bi_2_O_3_ of thickness *d*, respectively; d is the thickness of the film; and μ is the linear attenuation coefficient of the GNRL/Bi_2_O_3_ film. μ can be obtained measuring $I_{0}$, I(d), and d. Rearranging Equation (1), the AE can be calculated by Equation (2):

(2) $$\mathrm{AE}\%=\frac{\left(I_{0}-I\left(d\right)\right)}{I_{0}}\times 100=\left(1-e^{-\mu d}\right)\times 100$$

For a given material, if an attenuation efficiency for the actual working conditions is required, the requisite d of the material can be determined by Equation (2).

### 2.5. Film Uniformity 

**Figure 4:**
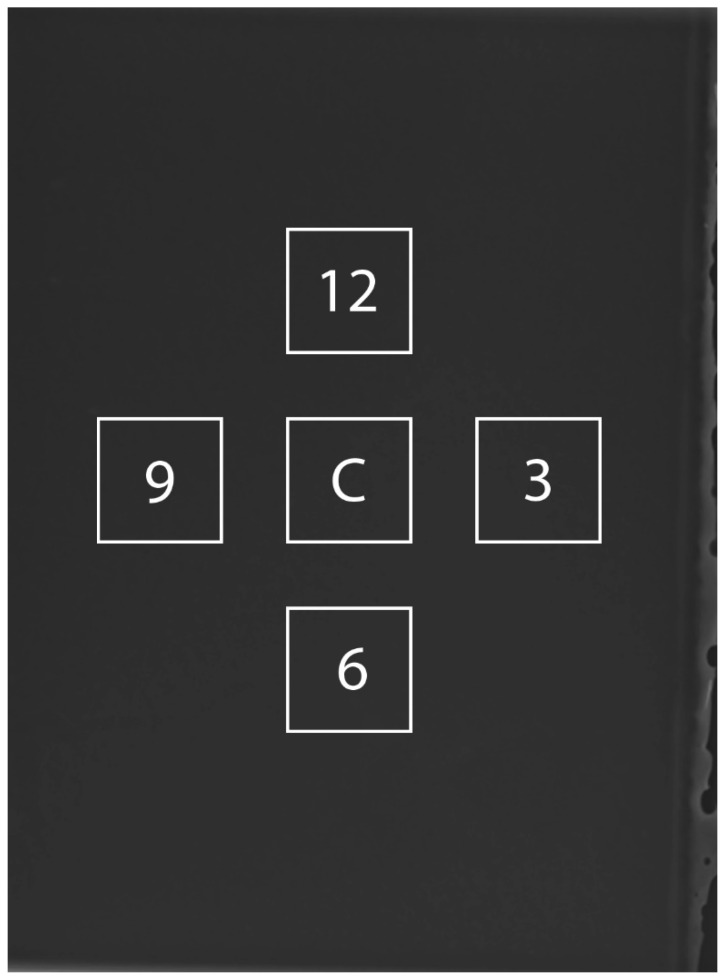
Illustration of film uniformity measurements. A square region of interest (ROI) of 100 × 100 pixels was placed at the image center. Additional ROIs were placed at radial positions of 3, 6, 9, and 12 o’clock. The center to center distance from each ROI is approximately 1 cm. Care was taken to avoid making radial measurements too close to the edge of the image.

Field uniformity was evaluated with the same digital radiography system used for radiation attenuation measurements. The source-to-image distance was set to 100 cm to minimize the non-uniformity of the X-ray beam. A lead apron was placed under the image receptor, and the film was placed on top of the receptor for contact mode imaging. The X-ray beam was collimated to the film, and 80 kVp and 1 mAs were used to achieve an exposure according to the radiography system manufacturer’s recommendations for detector response to minimize image noise. Raw images without any post-processing corrections were acquired. Regions of interest (100 × 100 pixels) were assessed in the film center and at each clock position of 3, 6, 9, and 12 (Figure 4). The film uniformity was calculated as a percentage of the mean pixel value for radial regions of interest relative to the center pixel value, as follows:

(3) $$Uniformity\;\left(\%\right)=\frac{\sum \left|P_{center}-P_{radial}\right|/n}{P_{center}}\times 100$$

$P_{radial}:$ Pixel value of radial regions of interest at 3, 6, 9, and 12 o’clock. 

$P_{center}:$ Pixel value of the center region of interest.

n: The number of radial regions of interest measured.

### 2.6. Mechanical Performance

**Figure 5:**
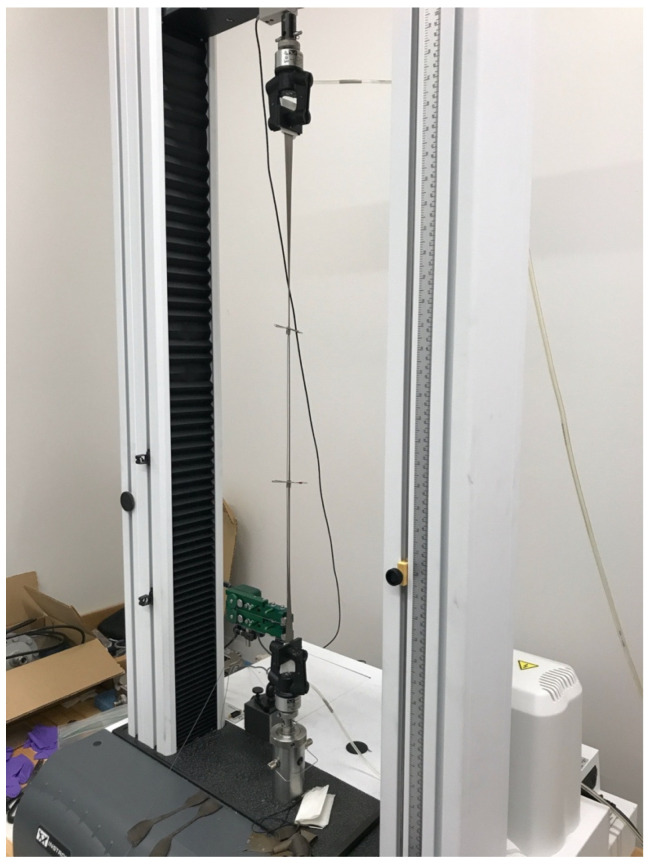
Mechanical testing of GNRL/micro-Bi_2_O_3_ samples by Instron universal testing machine.

Tensile measurements were performed according to ASTM D412 [51]. Five dumbbell specimens were cut using Die D (CCSI, Akron, OH, USA) from films, while forty-eight specimens were cut from gloves using Die C. Specimen thicknesses were measured in three places on the specimen across the testing area (at the center and both ends of the reduced section) and reported as the average. The tensile properties of the samples were determined using a tensiometer (model 3366, Instron, Norwood, MA, USA) with a 50 N static load cell (model 2530-50N, Instron), equipped with a contact extensometer (model 3800, Epsilon Tech. Corp., Jackson, WY, USA) (Figure 5). Three key parameters (tensile strength, ultimate elongation, and modulus at 500% strain) were derived from the raw data with the Bluehill v. 2.26 software package (Instron, Norwood, MA, USA).

### 2.7. Statistical Analysis

Statistical significances of the independent variables, i.e., filler loading, film thickness, and curing conditions, were determined by analysis of variance (ANOVA) with a significance level α of 0.05 using the software JPM software v.14 (SAS Institute Inc., Cary, NC, USA). Multiple means comparison Tukey–Kramer and Dunnett tests, at a significance level α of 0.05, were performed to determine statistical differences among film properties.

## 3. Results and Discussion

### 3.1. GNRL/Bi_2_O_3_ Film Thickness and Density

**Figure 6:**
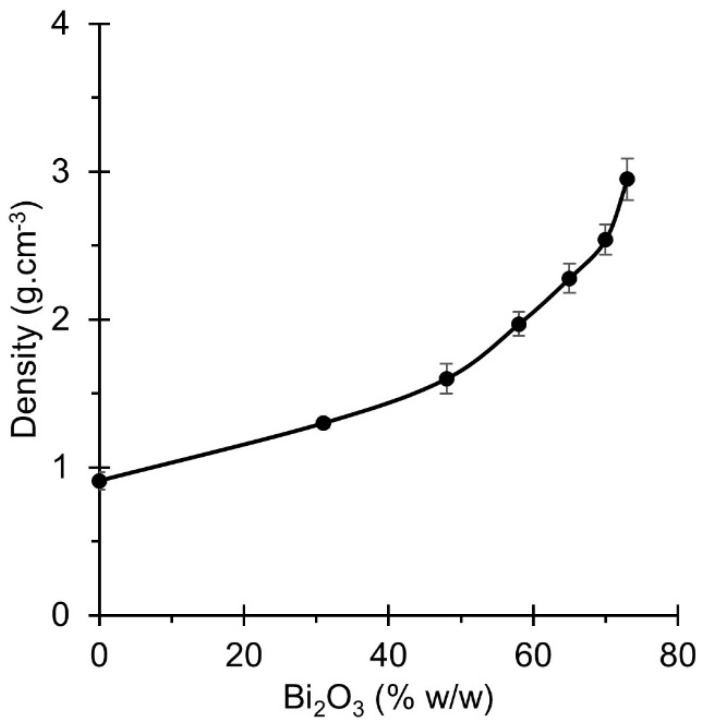
Density of the GNRL/micro-Bi_2_O_3_ films at different loadings of micro-Bi_2_O_3_. Results are expressed as means (*n* = 10) ± 1 s.d.

GNRL/Bi_2_O_3_ films from both casting and dipping methods ranged in thickness from 0.16 mm to 0.64 mm (Table 3) and 0.19 mm to 0.40 mm (Table 4), respectively. The density of the films was measured according to Archimedes’ principle to confirm the Bi_2_O_3_ fraction in the films. Film densities were plotted against mass fraction (Figure 6) and were in agreement with previous studies [47]. In general, the density of the films increased as the loading of the filler increased (Figure 6). This can be attributed to a higher density of Bi_2_O_3_ compared to the GNRL. The density did not have a linear relationship with Bi_2_O_3_ concentration in the films; rather, it seemed to increase with an exponential trend. X-ray AE increases when Bi_2_O_3_ concentration in the films increases [47]. Based on the results, an increase in the Bi_2_O_3_ concentration will significantly increase the density of films/gloves and thus their weight (Figure 6), while decreasing film/glove thickness (Table 3 and Table 4). Hence, the production of RAGs with a high concentration of micro-Bi_2_O_3_ must be carefully evaluated because the final weight may deter their use amongst HCWs.

### 3.2. Attenuation Properties of GNRL/Bi_2_O_3_ Films

The standard specification for radiation-attenuating gloves (ASTM D7866 [50]) establishes the minimum attenuation values at energy levels of 60, 80, 100, and 120 kVp as 29%, 22%, 18%, and 15%, respectively. Attenuation efficiencies (AE) and linear attenuation coefficients (*µ*) for films without and with Bi_2_O_3_ at X-ray peak energies of 60, 80, 100, and 120 kVp were calculated from Equation (2). The AE met or exceeded the attenuation standard for all films with Bi_2_O_3_, except for the 50 PHR films. The thinner films (0.22 and 0.24 mm) at 50 PHR Bi_2_O_3_ loading did not meet the standard at 60, 80, and 100 kVp, but were just below the limit at 120 kVp. The AE increased as film thickness and attenuating filler loading increased (data not shown).

**Figure 7:**
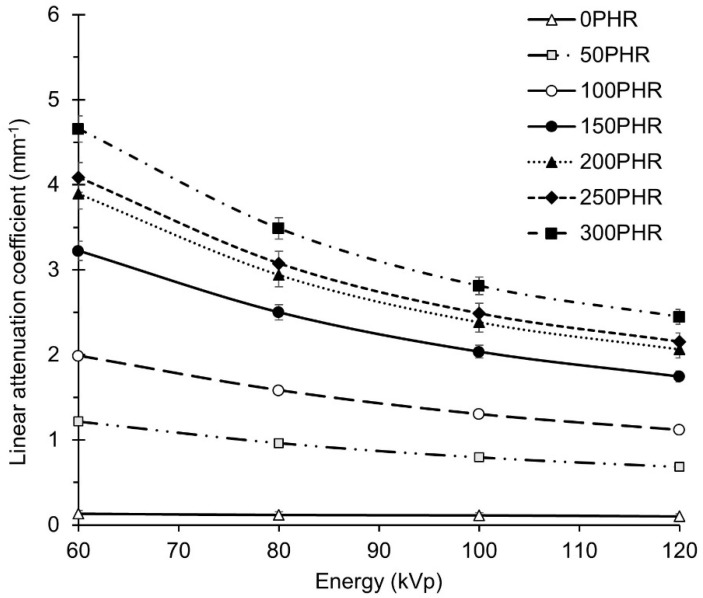
Linear attenuation coefficient (μ) at 60, 80, 100, and 120 kVp of GNRL films with 0, 50, 100, 150, 200, 250, and 300 PHR micro-Bi_2_O_3_. Results are expressed as means (*n* = 15) ± 1 s.d.

The linear attenuation coefficient, *µ*, for 0–300 PHR GNRL/Bi_2_O_3_ films was plotted against X-ray energy (Figure 7). Clearly, *µ* increased as filler loading in the GNRL matrix increased and diminished as energy level increased. The difference in the attenuation coefficient among films at the same energy became lower as filler loading increased. The differences started to diminish at micro-Bi_2_O_3_ loadings above 150 PHR (Figure 7). This could happen due to the agglomeration of the filler in those films with a high concentration of micro-Bi_2_O_3_ particles, thus producing films with a non-homogeneous dispersion of the filler [52]. Moreover, the difference in the attenuation coefficient among films at lower levels of energy is greater compared to those at higher energy levels because the photoelectric effect is predominant at low energy levels and Campton scattering increases at high energy levels [52]. Therefore, the results suggest that the production of RAGs with very high concentrations of micro-Bi_2_O_3_ may be impractical because filler agglomeration is expected and AE becomes progressively less additive at filler concentrations above 150 PHR. 

Existing radiation attenuation gloves on the market are formulated using HNRL (latex) or synthetic rubbers (latex-free) (Table 5). Attenuating fillers other than lead are now commonly used to avoid issues of lead toxicity in attenuation gloves [13,14,34]. The thickness of gloves on the market ranges from 0.18 mm to 0.35 mm, and the attenuation efficiencies (AE) are above those required by ASTM D7866 [50] (Table 5). However, the type and the concentration of the non-lead attenuation fillers used in these gloves on the market are not advertised in the published marketing literature. Moreover, the loading of the attenuating filler and the GNRL film thickness required to match and surpass the attenuation properties of currently marketed gloves and standards are unknown.

**Figure 8:**
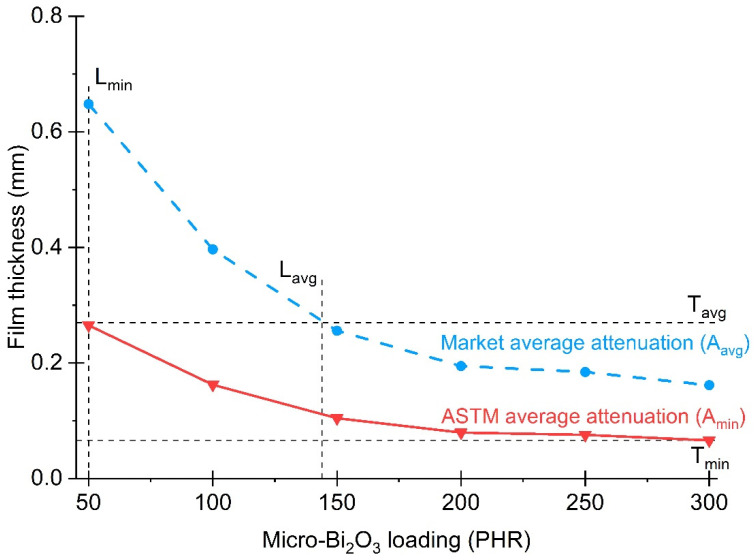
ASTM average attenuation and market average attenuation isocontour curves as a function of GNRL/micro-Bi_2_O_3_ film thickness and micro-Bi_2_O_3_ loading.

To predict these parameters for GNRL/Bi_2_O_3_ RAG, isocontour curves of constant attenuation were constructed as a function of film thickness and Bi_2_O_3_ loading (Figure 8). The thickness point (d) for each filler loading was obtained using Equation (2). The AE of marketed gloves and ASTM D7866 minimum required at each energy level is listed in the last two rows of Table 5. The μ for GNRL/Bi_2_O_3_ films at each energy level was previously calculated (Figure 7). The d values for each filler loading were obtained by averaging the values of d at each energy level.

The average loading of Bi_2_O_3_ (L_avg_) to achieve the market average attenuation (A_avg_) at the market average glove thickness (T_avg_ = 0.27 mm) was calculated using the market isocontour curve (Figure 8, blue line). The Bi_2_O_3_ L_avg_ at T_avg_ was 144 PHR. Likewise, the minimum average loading of Bi_2_O_3_ (L_min_) required to achieve the ASTM market average attenuation (A_min_) was 50 PHR at 0.26 mm and was calculated using the ASTM standard isocontour curve (Figure 8, red line). Additionally, the minimum thickness (T_min_) to meet the ASTM standard at the Bi_2_O_3_ loading (300 PHR) was 0.07 mm (Figure 8, lower horizontal dashed line). Thus, GNRL RAGs with excellent tactile sensitivity can potentially be achieved by reducing film thickness while maintaining the average attenuation above the A_min_ to comply with the standard. In this context, films produced during this study at a loading of Bi_2_O_3_ of 150 PHR and thicknesses of 0.20 mm and 0.27 mm, below and at the same T_avg_, respectively, achieved on average 9% less and 5% more, respectively, than the A_avg_ (Figure 8 and Table 5 and Table 6). Moreover, based on the results and isocontour curves, the model predicts that GNRL films with 150 PHR Bi_2_O_3_ loading and thicknesses of 0.15 mm and 0.30 mm, below and above T_avg_, respectively, can reach on average 19% less and 11% more than the A_avg_ (Figure 8 and Table 5 and Table 6). Therefore, as a first approach, an average loading of Bi_2_O_3_ of 150 PHR was used, and with this loading, a minimum thickness of 0.10 mm would be required for GNRL/Bi_2_O_3_ films to meet the ASTM standard (Figure 8). 

### 3.3. Optimization of Curing Conditions for GNRL/Bi_2_O_3_ Dipped Film 

Vulcanization conditions of GNRL/Bi_2_O_3_ films with 150 PHR Bi_2_O_3_ loading were previously reported [53]. In that study, the films were prepared by dipping, with curing temperature and times ranging from 70 °C to 105 °C at 5–10 °C intervals and from 35 min to 105 min at 5–15 min intervals, respectively, and their tensile properties were measured according to ASTM D412. Tensile properties showed that the best curing conditions tested were 90 °C and 75 min. The films cured with these conditions had modulus at 500% strain of 4.4 ± 0.7 MPa, tensile strength of 25.1 ± 0.2 MPa, and elongation at break of 788.0 ± 68.2%. These results demonstrated that GNRL/Bi_2_O_3_ films with 150 PHR Bi_2_O_3_ loading not only met attenuation standards (ASTM D7866) but also tensile properties for surgical gloves (ASTM D3577) and examination gloves (ASTM D3578) (Table 7). 

To further optimize the curing conditions, films made with 150 PHR Bi_2_O_3_ loadings were produced by the dipping method with a 10 s dwell time. Curing temperatures ranged from 87 °C to 96 °C at 3 °C intervals and curing time ranged from 65 min to 90 min at 5–10 min intervals. The tensile properties of the films were expressed as a ratio of films produced using the optimal curing conditions from the previous experiment (curing conditions of 90 °C and 75 min). Therefore, values above 1 represent an increase in the property over those films manufactured with the reference conditions, while values below 1 represent a decrease.

**Figure 9:**
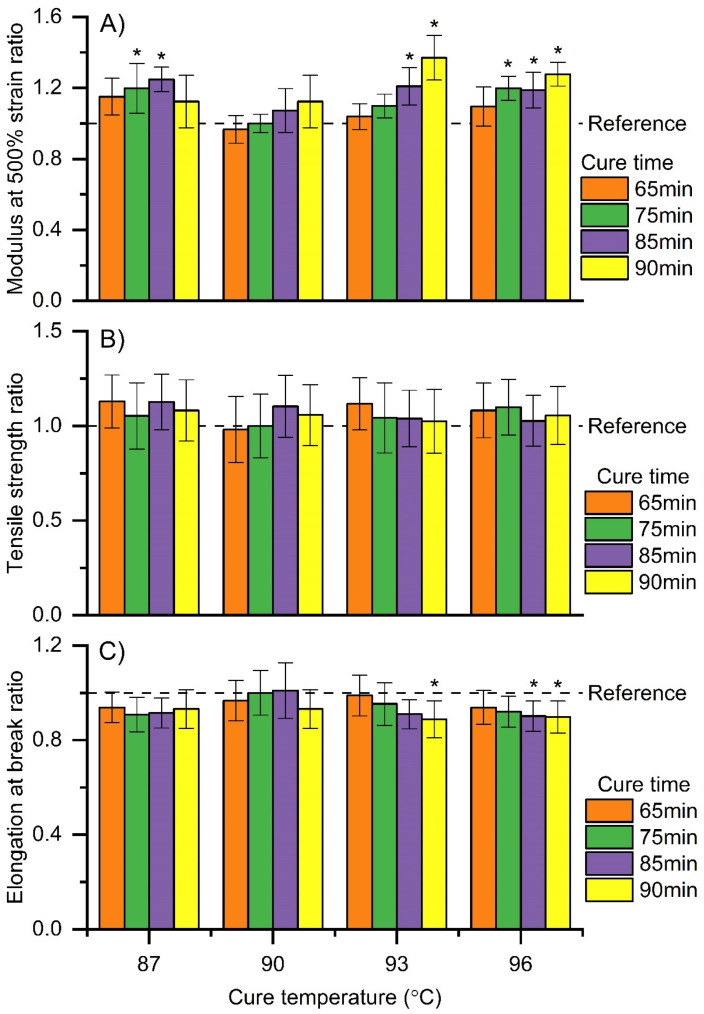
Ratios of mechanical performance of dipped films made with 150 PHR Bi_2_O_3_ versus reference films made with 150 PHR Bi_2_O_3_ loading and cured at 90 °C for 75 min as a function of curing temperature and different cure times. (**A**) Modulus at 500% strain ratio, (**B**) tensile strength ratio, and (**C**) elongation at break ratio. All films contained 150 PHR Bi_2_O_3_ and were dipped with a 10 s dwell time. Results are expressed as means (*n* = 10) ± 1 s.d. Treatments with tensile properties significantly different to the control group are marked with a star (*).

The film thickness was 0.24 ± 0.00 mm. The modulus at 500% strain significantly increased compared to the control when the curing temperature and time were 87 °C and 75–85 min, 93 °C and 85–90 min, and 96 °C and 75–90 min, respectively (Figure 9A). However, the standards call for a maximum modulus of 5.5 and 6.5 for surgical and exam HNRL gloves, respectively; hence, maximum deviations of 1.47 and 1.25 in the modulus from the control are allowed so films do not fail the D3577 and D3578 standards (Figure 9A). The results showed that cure conditions of 87 °C for 65 min, 90 °C for 85 min and 90 min, 93 °C for 65 min and 75 min, and 96 °C for 65 min can be used to produce acceptable films below the standard modulus limits.

Films with a relative tensile strength above 1 show an improvement over the control and are above the standard for surgical and exam gloves for tensile stress (24 and 18 MPa, respectively). However, tensile strength values below 0.96 and 0.82 do not meet the standards for surgical and exam gloves, respectively (Figure 9B). Therefore, although the tensile strength was not significantly different among the treatments and the control, it tended to improve (>1) with all curing conditions evaluated, except for films cured at 90 °C and 65 min (Figure 9B). 

In the case of elongation at break, films with relative values above 1 exhibit an improvement over the control and are above surgical and exam glove standards for tensile stress (750% and 650%, respectively). However, elongation values below 0.95 and 0.72 do not meet the standards for surgical and exam gloves, respectively (Figure 9C). The results show that the elongation at break was significantly lower than the control when the curing temperature and time were 93 °C for 90 min and 96 °C for 85–90 min, respectively. However, the curing condition of 90 °C for 85 min and 93 °C for 65 min tended to increase the maximum strain of the films, although it was not significantly different from the control (Figure 9C). 

In general, curing conditions of 90 °C for 85 min and 93 °C for 65 min improved all the tensile properties of the films, although the properties were not significantly different from the control. Curing conditions above 93 °C and above 75 min were detrimental. These conditions increased the modulus at 500% strain and reduced the elongation at break, making the films fail the ASTM medical glove standards. Further characterization using smaller temperature and time intervals should lead to the optimal curing protocol for this specific GNRL/Bi_2_O_3_ film. 

### 3.4. Effect of Bi_2_O_3_ Loading on GNRL/Bi_2_O_3_ Dipped Film Thickness and Tensile Properties

Films with 0, 50, 100, and 150 PHR micro-Bi_2_O_3_ were prepared by the dipping method with dwell times of 10, 20, 30, and 40 s for 0 PHR films and 3, 6, 10, and 20 s for the filled films. The curing condition for 0 PHR films was 75 min at 105 °C, and 75 min at 90 °C for the filled films. 

**Figure 10:**
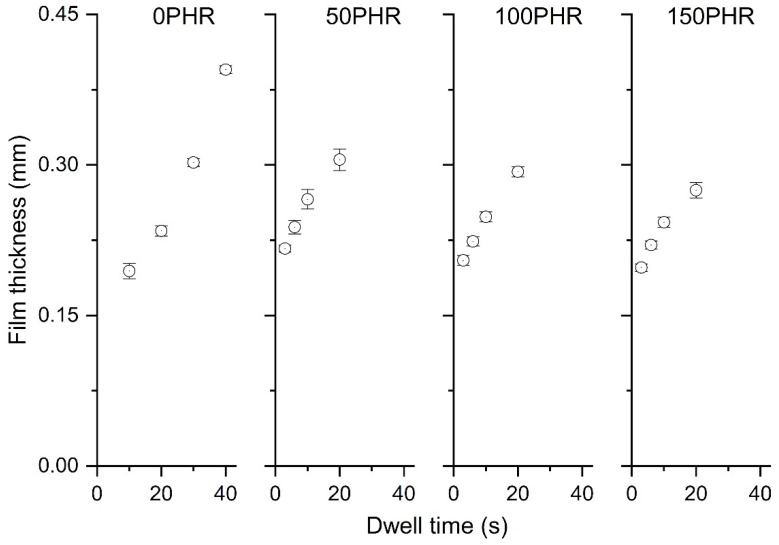
Film thickness vs. dwell time for films produced with 0, 50, 100, and 150 PHR. Results are expressed as means (*n* = 12) ± 1 s.d.

Film thickness ranged from 0.20 to 0.40 mm for 0 PHR films and from 0.20 to 0.30 mm for the filled films (Figure 10). Films containing Bi_2_O_3_ were thicker than those without filler produced at the same dwell time. Specifically, a 10 s dwell time generated 0.19 mm thick unfilled films and over 0.24 mm thick films with 50 PHR or greater Bi_2_O_3_ loading. The thickness of the filled films tended to decrease as the loading of Bi_2_O_3_ increased (Figure 10). Therefore, dwell times below 10 s are required to produce thinner films for filler loadings between 0 and 150 PHR.

**Figure 11:**
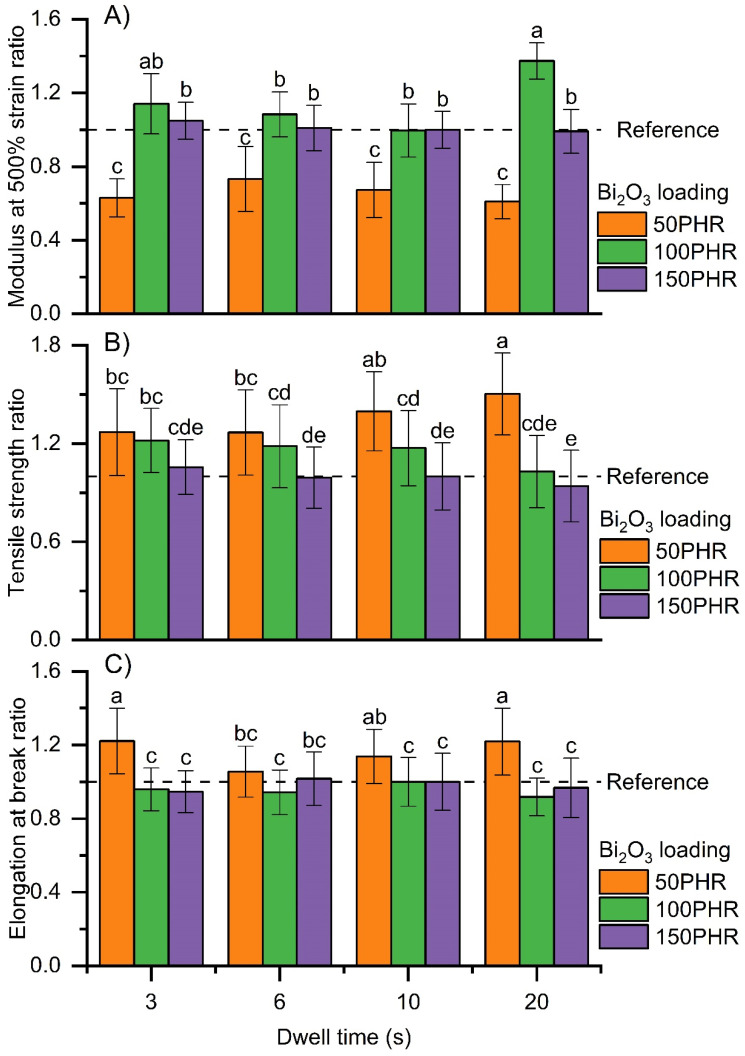
Effect of Bi_2_O_3_ loading and dwell time on ratios of mechanical performance of dipped films versus reference films made with 150 PHR Bi_2_O_3_ loading and cured at 90 °C for 75 min. (**A**) Modulus at 500% strain ratio, (**B**) tensile strength ratio, and (**C**) elongation at break ratio of dipped films with 50, 100, and 150 PHR Bi_2_O_3_ at dwell times of 3, 6, 10, and 20 s cured at 90 °C for 75 min versus reference films made with 150 PHR Bi_2_O_3_ loading. All films were cured at 90 °C for 75 min. Results are expressed as means (*n* = 10) ± 1 s.d. Films with statistically similar tensile properties are grouped together and marked with the same letter.

Tensile properties were evaluated among the treatments. Modulus at 500% strain was greater at 100 and 150 PHR attenuation filler than at 50 PHR filler within each dwell time (Figure 11A). The moduli were statistically the same for films with 100 and 150 PHR, except for films produced with 100 PHR filler and 20 min dwell time, which had the greatest modulus (Figure 11A). Tensile strength decreased as filler loading increased within each dwell time. Film strength increased as dwell time increased at 50 PHR filler but tended to decrease at higher filler loadings (Figure 11B). The elongation at break was greater for films with 50 PHR than films with 100 and 150 PHR within each dwell time. On the other hand, the elongation at break was not affected by dwell time (Figure 11C). 

The tensile properties were affected in different ways by filler loading and film thickness (which is directly correlated with dwell time). In general, filler loading had a greater effect on the tensile properties than did the film thickness. The decreasing performance properties of the GNRL/Bi_2_O_3_ composite films at high loadings is likely caused by the filler creating flaws within the composite material that prevents efficient stress transfer, leading to the reduction in tensile strength and ultimate elongation. This could be because high Bi_2_O_3_ loadings promote filler–filler interactions and lead to agglomeration. Additionally, weak filler–filler interactions (due to the non-polar nature of Bi_2_O_3_ [54]) are not effective at transferring stress.

The tensile properties of films made with 150 PHR Bi_2_O_3_ loading were similarly independent of the film thickness. These results suggest that, under the curing conditions used during this experiment, films with 150 PHR filler loading can be produced at thicknesses ranging from 0.20 mm to 0.27 mm without affecting their tensile properties.

### 3.5. Attenuation Uniformity

The RA uniformity was measured on films made with 150 PHR Bi_2_O_3_ loading and thickness ranging from 0.20 mm to 0.27 mm. The RA uniformity was greater than 96.6%, with a maximum value of 99.2% (Table 8). This demonstrates that the micro-Bi_2_O_3_ at 150 PHR loading was uniformly distributed in the films. 

### 3.6. GNRL RA Gloves

A total of sixty medium-sized GNRL RA gloves were individually produced at a glove thickness of 0.29 ± 0.02 mm. These GNRL/Bi_2_O_3_ gloves had modulus at 500% strain of 4.0 ± 0.9 MPa, tensile strength of 23.0 ± 4.4 MPa, and elongation at break of 798.0 ± 54.2%. GNRL RA glove values of modulus at 500% strain were below the HNRL surgical and examination glove standards maxima (Table 6). The tensile strength was consistently above the HNRL examination glove standard minimum and frequently above the surgical glove standard minimum (Table 7). The elongation at break value exceeded the HNRL surgical and examination glove standards (Table 7). These results demonstrate the potential of GNRL RA gloves to meet and surpass examination and surgical glove standards.

This study has determined and modeled the parameters for the development of new GNRL RA gloves. According to the results, ultra-thin and super-attenuating RAGs could be fabricated with excellent RA efficiencies. A GNRL RAG is being developed with 150 PHR micro-Bi_2_O to meet the attenuation requirements of ASTM D7866 [50] and the mechanical performance required in D3578 [16]. Thinner gloves than currently marketed RAGs (Table 5) also are being considered. These characteristics would provide an improved user experience with greater tactile sensitivity, leading to better patient outcomes. Likewise, a super-attenuating GNRL RAG can be formulated with 180 PHR micro-Bi_2_O and thicknesses above 0.27 mm to achieve RA efficiencies above marketed gloves while still meeting the examination glove performance requirements. It is expected that this super-attenuating GNRL RAG would improve tactile sensation over currently marketed RAGs. Additionally, surgeon’s GNRL RA gloves can be developed to meet the attenuation requirements of ASTM D7866 [50] and the mechanical performance required in D3577 [15] and/or D3578 [16]. A surgeon’s RAG with the same thickness as those in the marketplace (about 0.22 mm, Table 5) and 150 PHR micro-Bi_2_O_3_ loading would provide an attenuation level which matches or exceeds the maximum attenuations currently in the marketplace and provide improved user comfort and tactile sensation over existing products.

## 4. Conclusions

The different Bi_2_O_3_ loadings in the films could be linked to the variation in densities and thicknesses of the films. The AE increased as film thickness and attenuating filler loading increased. Isocontour plots indicated that GNRL RAGs could be made thinner than gloves currently in the marketplace, and that higher attenuation levels and user protection may be achieved without exceeding current RAG thicknesses. Films with filler loadings above 50 PHR showed AE above the standard specification for radiation-attenuating gloves (ASTM D7866). AE levels of GNRL/Bi_2_O_3_ films with 150 PHR Bi_2_O_3_ at RAG market average thickness were 5% more than the RAG market average attenuation. Furthermore, the films with 150 PHR Bi_2_O_3_ loading produced by optimized curing conditions were above the ASTM attenuation requirements and met the tensile requirements for natural latex examination and surgical gloves. Attenuation uniformity measurements in films with 150 PHR Bi_2_O_3_ loading demonstrated that the filler was uniformly distributed in the films. Therefore, RAGs produced by GNRL and Bi_2_O_3_ would eliminate the need for double-gloving during radiation-assisted procedures, further increasing user comfort and dexterity.

## 5. Patents

Cornish, K., Li, Z., Medical radiation attenuation medical glove, Application # 16/636,421—published (pending patent).

Cornish, K., Bioprocessing of Harvested Plant Materials for Extraction of Biopolymers and Related Materials and Methods, U.D. Patent 9873813.

## Figures and Tables

**Table 1:** Recipe for GNRL compounding.

Compound	Chemical	Dry wt (PHR)
GNRL	Guayule latex	100.0
Preservative	NH_4_OH	0.7
Antioxidant	Wingstay L	2.3
Activator	ZnO	0.5
Accelerator 1	ZDNC	0.9
Accelerator 2	DIXP	1.7
Sulfur	S	3.2

Key: PHR, parts per hundred of rubber; ZDNC, zinc di-isononyl dithiocarbamate; DIXP, di-isopropyl xanthogen polysulfide.

**Table 2:** Recipe for GNRL coagulant.

Component	Chemical	Loading
		% *w*/*w*
Coagulant	Ca(NO_3_)_2_·4H_2_O	25.4
Detackifier	Zn(C_18_H_35_O_2_)_2_	0.5
Surfactant	Triton X-100	0.5
Solvent	Water	72.9

**Table 3:** Prepared GNRL/Bi_2_O_3_ films by casting with different weight fractions (wt %) of attenuating filler and GNRL, amount of suspension added to the Petri dishes, and film thickness.

Bi_2_O_3_	Bi_2_O_3_	GNRL	Suspension	Film Thickness
PHR	% *w*/*w*	% *w*/*w*	g	mm
0	0	100	2.1	0.16 ± 0.01
			3.2	0.24 ± 0.03
			3.8	0.26 ± 0.02
			4.4	0.34 ± 0.02
			5.1	0.41 ± 0.02
			7.3	0.52 ± 0.03
150	58	42	3.9	0.16 ± 0.02
			5.8	0.22 ± 0.00
			7.8	0.31 ± 0.03
			9.6	0.39 ± 0.03
			11.5	0.49 ± 0.03
			12.9	0.57 ± 0.01
200	65	35	4.1	0.15 ± 0.03
			6.2	0.23 ± 0.03
			8.3	0.38 ± 0.03
			10.2	0.45 ± 0.04
			12.3	0.50 ± 0.04
			14.4	0.64 ± 0.05
250	70	30	4.3	0.20 ± 0.03
			6.6	0.31 ± 0.03
			8.7	0.38 ± 0.04
			11.0	0.46 ± 0.04
			13.0	0.56 ± 0.05
			15.0	0.63 ± 0.05
300	73	27	4.7	0.19 ± 0.03
			6.8	0.25 ± 0.03
			9.0	0.35 ± 0.04
			11.3	0.43 ± 0.04
			13.5	0.51 ± 0.04
			15.8	0.62 ± 0.04

**Table 4:** Prepared GNRL/Bi_2_O_3_ films by dipping with different weight fractions (wt %) of attenuating filler and GNRL, dwell time, and film thickness.

Bi_2_O_3_	Bi_2_O_3_	GNRL	Dwell Time	Film Thickness
PHR	% *w*/*w*	% *w*/*w*	s	mm
0	0	100	10	0.19 ± 0.01
			20	0.23 ± 0.01
			30	0.30 ± 0.00
			40	0.40 ± 0.00
50	31	69	3	0.22 ± 0.00
			6	0.24 ± 0.01
			10	0.27 ± 0.01
			20	0.30 ± 0.01
100	48	52	3	0.20 ± 0.00
			6	0.22 ± 0.00
			10	0.25 ± 0.00
			20	0.29 ± 0.01
150	58	42	3	0.20 ± 0.00
			6	0.22 ± 0.00
			10	0.24 ± 0.00
			20	0.27 ± 0.01

**Table 5:** Brand, thickness, attenuation efficiency (AE), and material of the radiation attenuation gloves on the market that are lead-free. Latex-free gloves are made of synthetic elastomers. AE is compared to the radiation-attenuating glove standard ASTM D7866 [50] minima.

Brand	Thickness	Attenuation				Material
		60 kVp	80 kVp	100 kVp	120 kVp	
	mm	%	%	%	%	
ASG	0.23	58	47	40	NA	Latex-free
Attenuator X	0.35	66	55	43	NA	Latex
IBG	0.18	56	47	41	38	Latex-free
Xguard RR1	0.22	45	35	26	23	Latex-free
Xguard RR2	0.30	55	45	35	31	Latex-free
Secure Touch XR1	0.20	46	36	30	24	Latex
Secure Touch XR2	0.35	64	54	48	42	Latex
Radiaxon latex	0.30	65	51	44	39	Latex
Radiaxon PI	0.30	62	53	46	40	Latex-free
Kiran NXPG35	0.35	63	53	46	41	Latex
Kiran NXPG25	0.25	51	44	39	27	Latex
Kiran NXPG20	0.20	41	33	28	21	Latex
**Average**	0.27	56	46	39	33	
ASTM D7866	n/a	29	22	18	15	

**Table 6:** Achieved and projected attenuation for films at 150 PHR Bi_2_O_3_ loadings and different thicknesses.

Bi_2_O_3_	Thickness	Attenuation				Results
Loading		60 kVp	80 kVp	100 kVp	120 kVp	
PHR	mm	%	%	%	%	
150	0.20	50	41	35	31	Achieved
150	0.27	57	48	41	36	Achieved
150	0.15	45	37	31	27	Projected
150	0.30	59	50	43	39	Projected

**Table 7:** Specifications for surgical/examination gloves.

Standard ASTM	Glove Type (Polymer Type)	Minimum Thickness	Minimum Tensile Strength	Minimum Ultimate Elongation	Maximum Modulus at 500% Strain
		mm	MPa	%	MPa
D3577	Surgical (HNRL)	0.1	24	750	5.5
D3577	Surgical (Synthetic)	0.1	17	650	7.0
D3578	Exam (HNRL)	0.08	18	650	6.5
D6977	Exam (CR)	0.05	14	500	-
D6319	Exam (Nitrile)	0.05	15	500	-

HNRL: Hevea natural rubber latex; CR: polychloroprene.

**Table 8:** RA uniformity of films made with 150 PHR Bi_2_O_3_ loading and thickness ranging from 0.20 mm to 0.28 mm. Regions of interest (100 × 100 pixels) were created in the center and at each clock position of 3, 6, 9, and 12 of the films.

Sample	Dwell Time	Thickness	Center	Mean Pixel Value from Raw Images				Uniformity
	s	mm		3	6	9	12	
1	3	0.20	4911.0	4704.0	4996.2	5132.4	4823.5	96.9%
2	3	0.20	4972.4	4790.8	4966.6	5210.0	4973.5	97.9%
3	3	0.20	4759.6	4596.5	4686.5	5017.9	4909.7	96.6%
4	6	0.22	4768.5	4660.0	4642.0	4930.2	4894.0	97.3%
5	6	0.22	4605.5	4521.4	4573.5	4776.2	4601.1	98.4%
6	6	0.22	5265.7	5196.4	5414.7	5466.5	5150.1	97.5%
7	10	0.25	5452.6	5412.0	5552.8	5562.3	5352.3	98.4%
8	10	0.24	4676.6	4656.5	4660.4	4769.4	4654.5	99.2%
9	10	0.24	5141.8	5144.4	5005.7	5271.4	5278.2	98.0%
10	20	0.28	5224.7	5099.8	5339.6	5515.8	5140.2	97.1%
11	20	0.27	4555.8	4390.2	4531.0	4786.7	4576.7	97.6%
12	20	0.28	4842.8	4752.2	4732.9	5138.2	4957.9	96.8%

## Data Availability

Data are available upon request from the corresponding author because the glove patent is still pending. The figures in this paper provide the results of the data.

## Nomenclature and Abbreviations

RAG	Radiation attenuation gloves
FDA	Food and Drug Administration
GNRL	Guayule natural rubber latex
PHR	Parts per hundred of rubber
AE	Attenuation efficiency
kVp	Kilovoltage peak
Bi_2_O_3_	Bismuth oxide
CT	Computed tomography
HCW	Heath care workers
NRC	Nuclear Regulatory Commission
ALARA	A slow as reasonably achievable
HNRL	Hevea natural rubber latex
Z	Atomic number
D	Particle size average
ZDNC	Zinc di-isononyl dithiocarbamate
DIXP	Di-isopropyl xanthogen polysulfide
SID	Source-to-image distance
*d*	Thickness of the film
I(d)	Peak intensity of the X-ray with a film of thickness d
$I_{0}$	Peak intensity of the X-ray without a film
μ	Linear attenuation coefficient
$P_{radial}$	Pixel value of radial regions of interest
$P_{center}$	Pixel value of the center region of interest
n	The number of radial regions of interest measured
L_avg_	Average loading of Bi_2_O_3_
A_avg_	Market average attenuation
T_avg_	Market average glove thickness
L_min_	Minimum average loading of Bi_2_O_3_ required to achieve the ASTM market average attenuation
A_min_	Market average attenuation
T_min_	Minimum thickness to meet the ASTM standard at the Bi_2_O_3_ loading 300 PHR
MPa	Mega Pascals
s.d.	Standard deviation
